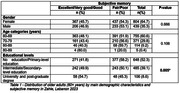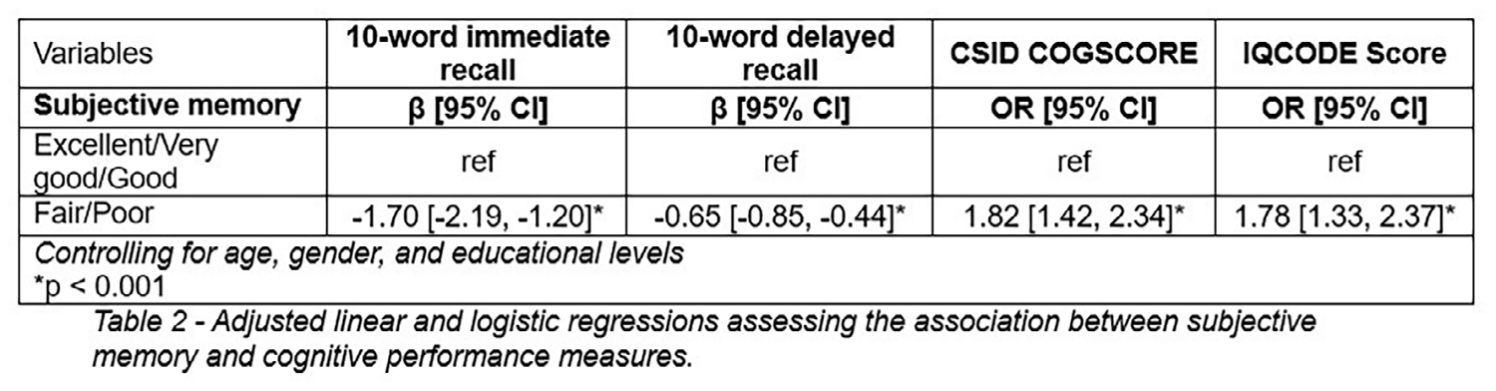# Subjective Memory and its Association with Cognitive Performance among Older Adults: Findings from a Cohort Study in Lebanon

**DOI:** 10.1002/alz.092253

**Published:** 2025-01-09

**Authors:** Monique Chaaya, Sawsan Abdulrahim, Martine Elbejjani, Stephen McCall, Aya El Sammak, Alexandra Abi Nassif, CH Henry Ngwa, Carlos Mendes de Leon

**Affiliations:** ^1^ American University of Beirut, Beirut Lebanon; ^2^ Faculty of Medicine, American University of Beirut, Beirut Lebanon; ^3^ American University of Beirut, Beirut, Beirut Lebanon; ^4^ Center for Research on Population and Health (CRPH), Beirut, Beirut Lebanon; ^5^ Georgetown University, Washington DC, DC USA

## Abstract

**Background:**

Rapid aging in low‐to‐middle income countries (LMICs) is expected to lead to exponential growth in dementia. As standard cognitive tools can be time‐consuming and cause fatigue for older adults, health professionals in primary care settings can utilize efficient measures to assess their cognitive performance. To date, there are still inconsistencies in the correlation between subjective memory complaints and cognitive impairment. Moreover, the validity of using a single‐item subjective memory is still not established. The aim of this study is to assess whether a one‐item measure of subjective memory is correlated with objective measures of cognitive performance among older adults in LMICs.

**Method:**

Lebanon Study on Aging and Health (LSAHA) is an ongoing population‐based cohort study of older adults aged 60 years or older, where 1,502 older adults were recruited from a rural district in Lebanon between October to December 2023. Cognitive performance was assessed using four tools: the Cognitive Domain in the ‘Community Screening Instrument for Dementia (CSI‐D)’ the ‘CERAD 10‐word’ immediate and delayed recall and the Informant Questionnaire on Cognitive Decline among the Elderly (IQCODE). Subjective memory was assessed by a single item and responses were categorized as: “good/very good/excellent” and “fair/poor.” Baseline data was analyzed using adjusted linear and logistic regression models to assess the association between subjective memory and cognitive performance measures.

**Result:**

Of the participants that responded to subjective memory and cognitive tools (n = 1,245), more than half (56.3%) reported fair/poor subjective memory. Adjusting for age, gender, and educational level, subjective fair/poor memory recall was significantly associated with higher odds of cognitive impairment based on CSID (OR:1.82 95%CI:1.42‐2.34) and IQCODE (OR:1.78 95%CI:1.33‐2.37) and a lower number of immediate (β:‐1.70 95%CI:‐2.19‐ ‐1.20) or delayed (β:‐0.65 95%CI:‐0.85‐ ‐0.44) word recall.

**Conclusion:**

This study’s findings suggest that a single‐item subjective memory assessment could be used by healthcare providers to identify older adults who may require further evaluation for cognitive impairment, similar to how self‐rated health has been a good marker for mortality and other health conditions. The results of this study warrant further research on this measure across different populations, comparing it with other validated screening tools.